# Compare mDCs and pDCs between two distinct patients groups in acute HIV-1 infection

**DOI:** 10.1186/1742-6405-11-22

**Published:** 2014-07-31

**Authors:** Yanmei Jiao, Xin Sun, Xiaojie Huang, Wei Li, Tong Zhang, Hao Wu

**Affiliations:** 1 Center for Infectious Diseases, Beijing You-an Hospital, Capital Medical University, Beijing 100069, China

**Keywords:** Acute HIV-1 infection, DCs, Rapid disease progression

## Abstract

The role of DCs in primary HIV-1 infection remains uncertain. In this study, we enrolled two different groups of subjects with acute HIV-1 infection. One group progressed to CD4 counts below 200 cells/μl within 2 years of HIV-1 infection (CD4 Low Group), while the other group maintained CD4 counts above 500 cells/μl (CD4 High Group). We did not find statistical difference in the pDC number between the two groups during acute HIV-1 infection. However, the mDC number was significantly lower in the CD4 Low Group than in the CD4 High Group.

## Introduction

Understanding how the innate immune response affects the outcome of HIV-1 infection in acute HIV-1 infection will open opportunities for vaccine development that can utilize the innate immunity to enhance viral control with minimal pathogenesis. Dendritic cells (DCs) are particularly important innate immune cells and HIV-1 exploits DCs to enhance infection. Thus, DCs are a critical link between virus, CD4+ T-cells, and CD8+ T-cells. DCs are divided into two broad subsets, myeloid (mDC) and plasmacytoid (pDC), based on phenotype, function, and tissue localization. Although details of these subsets are debated and vary based on species, pDCs are specialized early type 1 interferon-secreting cells that initiate antiviral adaptive immune responses. mDCs differentiate from immature bone marrow (BM)-derived precursors and function as peripheral sentinels by transmitting antigen derived signals to draining lymph nodes (LN). mDCs secrete high levels of interleukin-12 (IL-12) and are key players in amplifying adaptive immune responses [1]. Early immune events during HIV infection are associated with the rate of subsequent disease progression. A role for DCs in controlling HIV-1 replication during primary infection has been difficult to assess, given the difficulties in finding individuals with acute HIV infection. The aim of this study is to study the relationship between DCs number in acute infection and disease progression.

## Materials and methods

### Patients

35 patients recently infected with HIV-1 were recruited from an HIV-1-negative high-risk MSM (men who have sex with men) cohort. They were screened every 2 m for HIV-1 infection from October 2006 in the Beijing You’an Hospital [2]. Thirteen of the 35 patients showed rapid progression of HIV-1 disease, with CD4 counts < 200 cells/ul within 2 y post-infection (CD4 Low Group), while 22/35 cases enrolled in the study maintained a CD4 count higher than 500 cells/ul (CD4 High Group). The progression of early HIV-1 infection can be depicted as six discrete stages, as proposed by Fiebig et al. [3]. All the 35 enrolled patients were in Fiebig stage III. The project was reviewed and approved by the Beijing You’an Hospital Research Ethics Committee, and patients participated in the study following informed consent. Demographic and immunologic characteristics of the patients are reported in Table 1.

**Table 1:** Characteristics of patients in this study

**Patient**	**Age**	**Initial CD4 count**	**Last CD4 count**	**Initial VL**	**VL set point**	**Days from the initial positive point to CD4 < 200 cells/ul**
	**(year)**	**(cells/ul)**	**(cells/ul)**	**(copies/ml)**	**(copies/ml)**	
1	22	614	181	1,558	30,800	714
2	23	296	159	8,690	24,600	459
3	23	314	188	53,000	28,400	196
4	25	327	171	110,000	79,600	169
5	26	415	117	392,000	153,600	153
6	26	64	117	26,900,000	714,000	172
7	27	349	153	61,400	61,400	218
8	29	265	118	412,000	393,000	189
9	30	610	72	9,490	7,090	755
10	32	296	145	400,000	26,000	260
11	34	499	69	252,000	776,000	191
12	36	285	53	13,300	13,300	345
13	43	130	195	16,200	11,940	356
14	22	792	605	70,200	662	━
15	23	598	714	34,000	9,700	━
16	23	716	527	14,100	7,210	━
17	24	805	827	56,800	35,900	━
18	24	603	689	16,400	527	━
19	25	552	865	9,170	1,040	━
20	25	716	530	14,900	1,940	━
21	26	678	622	1,440	3,260	━
22	26	823	521	15,000	2,000	━
23	26	805	683	258,000	61,200	━
24	27	640	619	15,500	4,530	━
25	29	716	546	8,780	8,390	━
26	30	813	790	9,700	2,300	━
27	30	745	589	8,260	1,500	━
28	31	823	648	809	200	━
29	32	678	546	18,500	5,200	━
30	32	1148	1056	1,030	554	━
31	34	558	538	26,500	9,700	━
32	34	835	546	10,050	1,890	━
33	37	562	568	27,600	7,960	━
34	38	792	784	70,200	1,312	━
35	40	720	639	6,200	3,320	━

VL: viral load.

### Flow cytometric analysis

**Figure 1:**
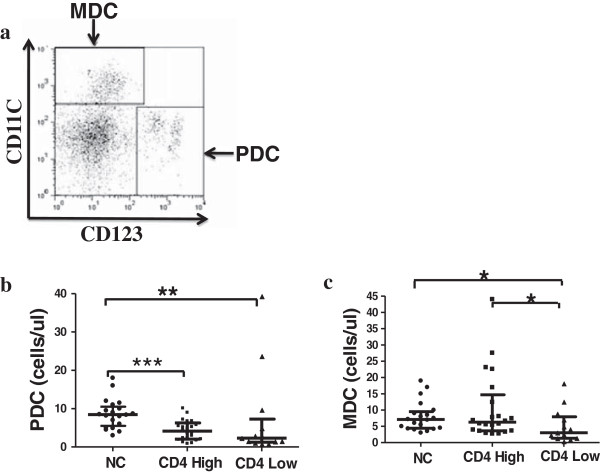
**Comparison of DCs between the three groups. (a) **Analysis of pDC and mDC by flow cytometry, Comparison pDC **(b)** and mDC **(c)** number between normal control and CD4 High Group and CD4 Low Group. Bars indicate median with interquartile range. ***p < 0.001, **p < 0.01, *p < 0.05.

To identify DCs, the following antibodies from BD Pharmingen (San Diego, CA, USA) were used: Lin-FITC, CD123-PE and CD11c-APC. At least 200,000 events were acquired for each sample. mDCs were identified as Lin-CD123-CD11c+, while pDCs were Lin-CD123 + CD11c-(Figure 1a). DC counts were calculated as follows, using hemocytometer data for lymphocytes and monocytes and flow cytometry data for DC windows, as described previously [4,5].

Absolute blood CD4+ T-cell counts were measured using a FACSCalibur flow cytometer (BD, Franklin Lakes, NJ, USA). Viral load was measured by the Amplicor (Roche Diagnostic Systems, Indianapolis, IN, USA) HIV-1 monitor ultrasensitive method with a detection limit of 40 copies/mL of plasma.

### Assays for plasma HIV-1 RNA

Plasma HIV RNA was quantified by real-time PCR (Roche, Germany), a super-sensitive method. The sensitivity of detection of this assay was 40 copies/ml.

### Statistical analysis

Comparisons were performed using the nonparametric independent sample tests, and all reported p values were two-sided and considered significant at p < 0.05. All data were analyzed using SPSS statistical software (version 16.0; SPSS, Chicago, IL, USA).

## Results

To study the relationship between DCs and disease progression, we compared the pDC and mDC number in Fiebig stage III between the CD4 High, CD4 Low, and normal control groups. We found a higher pDC number in normal controls compared with the CD4 High and CD4 Low groups (Figure 1b). The pDC number between the CD4 High and the CD4 Low groups did not differ significantly (Figure 1b). However, mDCs were significantly lower in the CD4 Low relative to CD4 High and normal controls (Figure 1c). There was no statistically significant difference in the mDC number between the CD4 High and normal controls (Figure 1c). DC numbers were negatively correlated with HIV viral load (Table 2).

**Table 2:** Results of spearman correlation analysis

	**Viral load**	**Viral load set point**
pDC	-0.323*	-0.350*
mDC	-0.233	-0.282

Correlation coefficients (Spearman correlation analysis) are shown.

*P < 0.05.

## Discussion

Our results are consistent with reports that DCs are markedly reduced in number during acute HIV-1 infection [6-9], particularly pDCs. The mechanism behind the decline in pDC numbers in acute HIV infection is not clear. It could be because of apoptosis as a direct result of infection [10,11] or mediated by TRAIL and Fas ligand–Fas interactions; it could be a consequence of compromised production of pDC precursors because of bone marrow infection; or it may reflect pDC migration to lymphoid tissues after HIV-induced activation.

mDCs express apolipoprotein B mRNA editing enzyme catalytic polypeptides (APOBECs), proteins that deaminate cytidine to uridine in nascent minus-strand viral DNA, blocking HIV replication [11,12]. Mature mDCs increase APOBECG expression, explaining their relative resistance to HIV-1 infection. mDCs capture and process HIV-1, and present associated antigens to T-cells. Thus, the loss of mDCs may on the one hand decrease APOBECG expression. On the other hand, the loss of mDCs decrease their ability of capture and process HIV-1 and present associated antigen to T cells. Therefore, this may explain why the loss of mDC in acute HIV infection could lead to rapid disease progression.

In conclusion, we found that the loss of mDC rather than pDC from the blood during acute HIV infection is associated with rapid disease progression. However, key questions remain to be answered regarding tissue distribution, development, and functional regulation.

## Competing interests

The authors declare that they have no competing interests.

## Authors’ contributions

YJ drafted the manuscript and statistical analyses. XS participated in flow cytometric analysis. TZ followed up patients and collected samples. XS and XH assisted with manuscript and data anlysis. YJ and WL assisted with flow cytometric analysis and data acquisition. HW conceived the study and participated in the data analysis. HW supervised and coordinated the study. All authors have read and approved the final manuscript.
